# A Novel Missense *WFS1* Variant: Expanding the Mutational Spectrum Associated with Nonsyndromic Low-Frequency Sensorineural Hearing Loss

**DOI:** 10.1155/2022/5068869

**Published:** 2022-10-03

**Authors:** Jingyu Ma, Rongrong Wang, Li Zhang, Shanshan Wang, Shuqing Tong, Xiaohui Bai, Zhiming Lu

**Affiliations:** ^1^Department of Laboratory Medicine, Shandong Provincial Hospital Affiliated to Shandong First Medical University, Jinan, China; ^2^Department of Laboratory Medicine, Shandong Provincial Hospital, Shandong University, Jinan, China

## Abstract

**Background:**

Nonsyndromic low-frequency sensorineural hearing loss (LFSNHL) is an uncommon form of hearing loss (HL) that typically affects frequencies at 2000 Hz and below. Heterozygous variants in the *WFS1* gene at the DFNA6/14/38 locus are considered a common cause of LFSNHL. To date, 34 different pathogenic genetic variants have been reported to cause LFSNHL with seven of these variants identified in the Chinese population. However, limited reports are available on the association between *WFS1* gene and LFSNHL. Here, we report a five-generation Chinese family with an autosomal dominant inheritance pattern of postlingual and progressive LFSNHL.

**Methods:**

Routine clinical and audiological examinations were performed on 16 affected and 7 healthy members in this family. The targeted next-generation sequencing of 127 known deafness genes was performed to identify variants in affected individuals. Sanger sequencing were further employed to confirm the pathogenic variant identified.

**Results:**

A novel heterozygous pathogenic genetic variant c.2530G > T (p.Ala844Ser) was identified in the *WFS1* gene in all patients of this family. The mutated Ala residue is evolutionarily conserved and cosegregated with HL. The variant was predicted to be deleterious by MutationTaster, PolyPhen-2, LRT, and Fathmm software. Conservation analysis and 3D protein structure model indicated that the variant caused a structural change in the protein.

**Conclusions:**

Our present study identifies a novel heterozygous *WFS1* variant associated with LFSNHL in a Chinese family.

## 1. Introduction

Hearing loss (HL) is the most common sensory impairment, severely affecting people's lives [1]. According to the World Health Organization report, more than 1.5 billion people worldwide have varying degrees of HL, which is expected to increase to more than 2.5 billion by 2050 (https://www.who.int/zh/news-room/fact-sheets/detail/deafness-and-hearing-loss), generating heavy social and economic burdens on many individuals and families as well as the society. There are numerous factors that cause deafness, such as aging, ototoxic drugs, environmental factors, genetic factors, inflammation, and other unknown causes [2]. However, it is worth noting that more than 60% of severe HL cases in neonates or early childhood are related to genetic factors [1]. Depending on its association with other organ abnormalities, the cases of hereditary deafness are generally grouped into two categories, i.e., syndromic HL and nonsyndromic hearing loss (NSHL), accounting for 30% and 70% of the total cases of hereditary deafness, respectively [3]. The NSHL inheritance patterns include autosomal recessive (80%), autosomal dominant (15-20%), sex chromosome chain disorders (1%), and mitochondrial DNA inheritance (1%) [4]. To date, a total of 161 genetic loci (68 dominant and 93 recessive) and 134 genes (45 dominant, 74 recessive, and 5 X-linked) have been reported to be associated with NSHL based on the Hereditary Hearing Loss database (https://hereditaryhearingloss.org/; accessed in August 2021).

The low-frequency sensorineural hearing loss (LFSNHL) is a rare type of HL that affects low frequencies of 2000 Hz and lower, showing a characteristic audiogram configuration that is mostly ascending. Studies have shown that a group of four genes, including *DIAPH1*, *MYO7A*, *CCDC50*, and *WFS1*, are associated with the nonsyndromic LFSNHL [5–7]. The variants in *WFS1* are considered common causes of LFSNHL [8, 9]. For example, Fukuoka et al. reported that variants in the *WFS1* gene were detected in one-third autosomal dominant LFSNHL Japanese families during the screening of 206 Japanese autosomal dominant and 64 autosomal recessive (sporadic) nonsyndromic HL probands [8].

Hearing screening combined with genetic diagnosis can help us detect more pathogenic genetic variants. In this study, we performed mutational analysis of a five-generation Chinese family using both deafness genes NGS panel and Sanger sequencing techniques. The missense variant *WFS1* c.2530G > T (p.Ala844Ser) was identified as the cause of this nonsyndromic LFSNHL in this family. Four pathogenicity predicting tools were used to indicate that the variant was deleterious, and 3D protein structure model predicted showed that the novel variant in WFS1 may alter the normal structure of the wolframin protein. Our findings would be a significant addition to the collection of *WFS1* variants and would make the genetic counseling available for this family.

## 2. Materials and Methods

### 2.1. Study Subjects and Controls

The family with LFSNHL comprised a total of 94 individuals in five generations, including 26 affected members. With the informed consent obtained from each individual, a total of 23 family members (16 affected and 7 unaffected) participated in a clinical evaluation study. A total of 200 individuals with normal hearing were selected as controls. The written informed consents of all participants were obtained before their inclusion in the study, which was approved by the Ethics Committee of Shandong University (approval number 014).

### 2.2. Clinical Data

A medical history was obtained from each participant using a questionnaire to collect the following data: age at onset, evolution, and symmetry of the hearing impairment, presence of tinnitus, use of aminoglycoside antibiotics, and other relevant clinical manifestations. All participants were given the clinical and audiological evaluations generally performed in the physical examination, including pure-tone audiometry (PTA), acoustic impedance, tympanometry, auditory brainstem response (ABR), distortion product otoacoustic emission (DPOAE), electrocochleogram, and vestibular function. Blood glucose test and optic nerve electroretinogram were performed to verify other complications other than hearing disorders in the family members. Environmental factors, such as acoustical noise, were excluded as causes of HL. Severity of HL was classified by the average value of PTA at 500, 1,000, 2,000, and 4,000 Hz as mild (26–40 dB HL), moderate (41–55 dB HL), moderately severe (56–70 dB HL), severe (71–90 dB HL), and profound (>90 dB HL), respectively.

### 2.3. DNA Extraction

Genomic DNA was extracted from 2 mL peripheral blood with AxyPrep Genomic Blood DNA Extraction Kit (AXYGEN, Corning, USA) in each of the 23 members of this family (i.e., II-7, III-6, III-8, III-10, III-11, III-14, III-16, III-20, III-22, IV-2, IV-8, IV-10, IV-11, IV-12, IV-13, IV-14, IV-22, IV-29, V-1, V-3, V-8, V-9, and V-15) and a total of 200 normal controls.

### 2.4. Targeted Deafness Gene Capture and Next-Generation Sequencing

Targeted genomic capturing and next-generation sequencing analyses were performed on proband (III-10) by BGI Inc. (Shenzhen, China). All exons, splicing sites, and immediate flanking intron sequences of a total of 127 deafness genes (Supplementary Table 1) were sequenced. The E210 DNA-shearing instrument (CovarisS2, Massachusetts, USA) randomly fragmented genomic DNA samples into fragments of 200-300 base pairs. Then, the end-repair, adenylation, and adapter ligation were performed for library preparation following the standard Illumina protocols. Capture of targeted DNA fragments was conducted by hybridization with capture arrays. High-throughput sequencing for captured library was performed on Illumina HiSeq2000 Analyzers. Three-step filtering analyses were performed on the raw data to generate “clean reads” for further analysis. First, the indexed primers were used to identify different reads from different samples in the primary data (i.e., reads that were perfectly matched to the theoretically indexed sequences and reads that were matched with the theoretical primer indexed sequences with a maximum of three mismatches). Second, the reads containing partial adapter sequences were removed. Third, the low-quality reads (i.e., reads containing more than 10% Ns in the read length as well as 50% reads with a quality value of less than 5 and with an average quality of less than 10 and adapter sequences including indexed sequence) were removed [10]. The high-quality clean reads were aligned to the human reference genome (hg19) using the BWA (Burrows-Wheeler Aligner) MultiVision software package. SOAPsnp software and GATK Indel Genotyper were used to identify SNPs and indels, respectively. The novelty of variant was determined by screening of 1000 Genomes, HapMap, dbSNP, gnomAD, and the BGI in-house databases. The pathogenic features of missense variant were predicted by several different computer algorithms (i.e., MutationTaster, PolyPhen-2, LRT, and Fathmm). We excluded variants with minor allele frequencies >0.05 in the 1000 Genomes Project and PVFD database (https://github.com/BGI-flexlab/PVFD). Pathogenicity of the variant was classified based on the guidelines of the American College of Medical Genetics and Genomics (ACMG) 2015 [11].

### 2.5. Sanger Sequencing

Sanger sequencing of *WFS1* was performed using the sequence specific primers, i.e., forward 5′-GTTCAAGAGCGTGCTGCTCA-3′ and reverse 5′-AGTCGAAGGCGAACTTCACG-3′ for c.2530G > T variant. Data analysis was performed using Chromas2.4.1 software. The *WFS1* gene sequence (NM_001145853.1) and WFS1 protein sequence (NP_001139325.1) were used as the references for sequence alignment.

### 2.6. Structure Modeling

Protein structures of wild-type and mutant WFS1 (NP_001139325.1) were modeled using I-TASSER (https://zhanglab.ccmb.med.umich.edu/I-TASSER/). The protein sequences were submitted in the query box in FASTA format. The PDB files obtained from I-TASSER server were analyzed using PyMOL software.

### 2.7. Cell Culture

The human embryonic kidney cells (HEK-293) were cultured with Dulbecco's modified Eagle's medium (DMEM, Gibco, USA) containing 10% fetal bovine serum (FBS, Gibco, USA), 100 U/mL penicillin, and 100 *μ*g/mL streptomycin at 5% CO_2_ and 37°C. The House Ear Institute-Organ of Corti 1 (HEI-OC1) cell line was maintained in DMEM with 10% FBS at 10% CO_2_ and 33°C.

### 2.8. Plasmid Construction and Immunofluorescence

The plasmids used to load *WFS1* cDNA was obtained from BioSune Biotech (Jinan, China). The cDNA sequence was confirmed by using Sanger sequencing. These *WFS1* cDNA-loaded plasmids were then used to amplify wild-type (WT) *WFS1* by PCR. The PCR primers for *WFS1* cDNA region were as follows: 5′- ACGGTACCATGGACTCCAACACTGCTCCGCTGGGC-3′ and 5′- ACGCGGCCGCTCACTTGTCATCGTCGTCCTTGTAGTCGGCCGCC-3′. The PCR products were digested by KpnI and NotI and cloned into p3 × FLAG-Myc-CMV-24 vector (Sigma). To construct the plasmids containing mutated *WFS1*, the ClonExpress Entry One Step Cloning Kit (Vazyme) was used to introduce the variant (c.2530G > T) into the WT vector. Both HEI-OC1 and HEK-293 cells were plated in 24-well plates, respectively, then transfected with 1.0 *μ*g of *WFS1* WT or mutant plasmids using Lipofectamine 3000 transfection reagent (Invitrogen, Carlsbad, CA, USA). In 24 h, cells were fixed in 4% paraformaldehyde/PBS for 10 min, permeabilized by adding 0.1% Triton X-100 for 1 min, and then blocked with PBST containing 1% donkey serum at 37°C for 1 h. The cells were then incubated with primary anti-FLAG antibody (1 : 100, 8146, CST, USA) and anti-Calnexin antibody (1 : 100, ab224465, Abcam, USA) overnight at 4°C. Then, the secondary goat antimouse, goat antirabbit (Abcam), and DAPI antibodies (D9542, Sigma-Aldrich, US) were incubated with cells at room temperature for 1 h. After sealing, the immunofluorescence imaging was performed by a Leica TCS SP8 confocal fluorescence microscope (Leica Microsystems, Biberach, Germany).

## 3. Results

### 3.1. Clinical Features of the Participants

**Figure 1:**
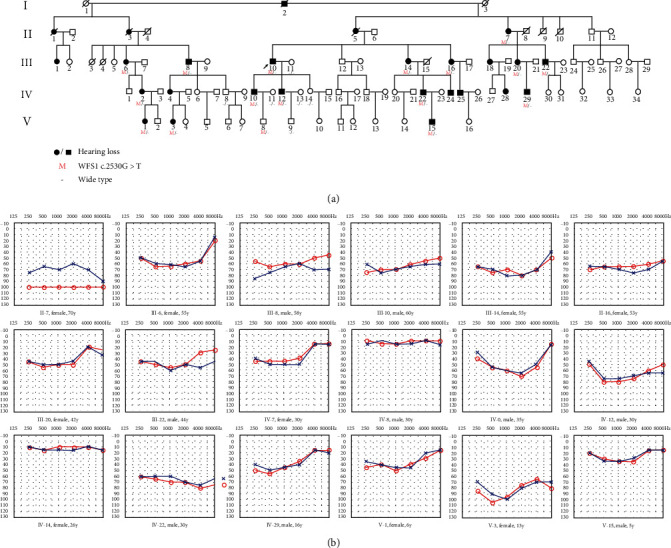
Pedigree of the 5-generation Chinese Han family and their audiological phenotypes. (a) Pedigree of the family affected with nonsyndromic hearing loss showing an autosomal dominant hereditary pattern. Males are denoted by squares, females by circles, and deceased by a diagonal line through the symbol. Proband: arrow; white symbol: normal hearing; black symbol: hearing impairment. (b) The pure-tone audiograms of some representative members in this family. Frequency in hertz (Hz) is plotted on the *x*-axis and the auditory threshold in decibels (dB HL) on the *y*-axis.

**Figure 2:**
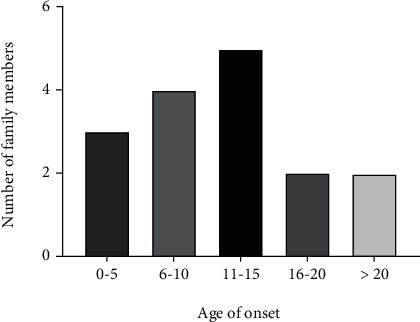
Age (increment of 5 years) of onset of hearing loss in the members of the 5-generation Chinese family.

A five-generation Chinese family with a total of 94 members from Shandong Province, China, participated in this study with the pedigree constructed according to the verbal descriptions of the participants (Figure 1(a)). The affected members of this Han Chinese family were presented with dominant NSHL, showing bilateral, symmetric, and progressive hearing impairment. The PTA results showed that it was predominantly low-frequency impaired (Figure 1(b)). In addition, according to the age distribution map, the age at onset of hearing impairment was varied from 1 to 30 years in this family, and most of these patients mainly developed HL around 10 years old (Figure 2). The proband (III-10) developed severe sensorineural HL involving low frequencies; the average auditory thresholds on PTA for the right and left ears were 67 and 63 dB HL, respectively (Figure 1(b)). Only wave V was observed in both ears under 96 dB HL stimulation on ABR testing, and DPOAE were absent bilaterally.

Results of MRI, vestibular function tests, electrocochleography, and utricle and balloon function tests revealed no abnormality of otology-related malformations or vestibular dysfunction in the participants. The acoustic immittance tests were all A-type tympanograms, and the external auditory canal volume, tympanic pressure, and compliance values were all normal. All family members appeared normoglycemic. Assessment of optic nerve electroretinograms and fundus images revealed no abnormalities. A comprehensive clinical examination and medical histories revealed no other clinical syndrome phenotypes. The main clinical features of this family are described in Table 1.

### 3.2. Detection and Analysis of Variants

**Figure 3:**
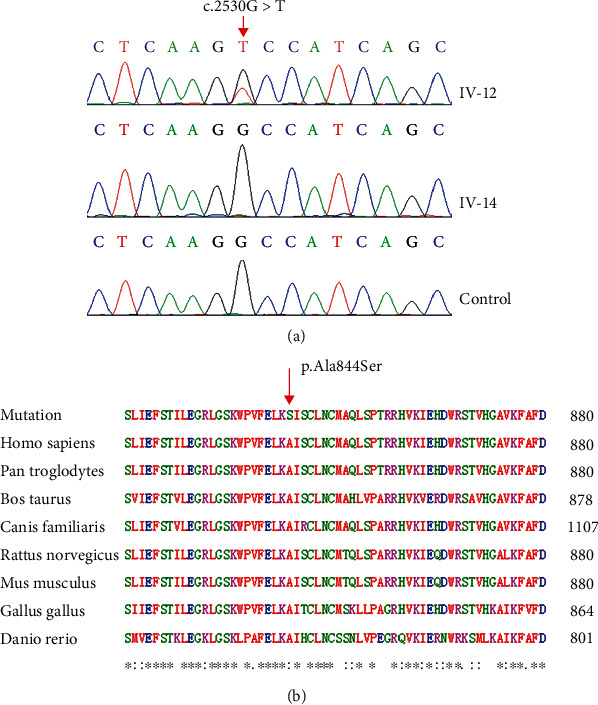
Sequence chromatograms, conservation analysis of *WFS1* (a) Chromatograms showing the c.2530G > T variant in the 5-generation Chinese family with LFSNHL. Partial sequence chromatograms of *WFS1* gene from the affected individual IV-12, the normal-hearing individual IV-14 of the family, and a control. The red arrow indicates the location of the nucleotide changes at position 2530. (b) Protein alignment shows highly conserved nature of the p.Ala844 residue (indicated by a red arrow) in patients in our study and other 8 sequences from *Homo sapiens*, *Pan troglodytes*, *Bos taurus*, *Canis familiaris*, *Rattus norvegicus*, *Mus musculus*, *Gallus gallus*, and *Danio rerio*.

In order to identify the pathogenic variant in this family, the targeted gene capture sequencing based on a total of 127 genes associated with nonsyndromic and syndromic deafness was performed in the proband (III-10). Six variants (*ATP2B2*, *WFS1*, *SERPINB6*, *TRIOBP*, *OTOA*, and *MYH9*) were detected to be possible candidates. After removing the synonymous variants, the heterozygous variant c.2530G > T (p.Ala844Ser) in *WFS1* was identified as the only candidate variant consistent with the autosomal dominant inheritance patterns in this Chinese family, i.e., the heterozygous state of *WFS1* variant can cause HL in affected family members. Then, the Sanger sequencing was performed in all participants of the family and 200 controls with normal hearing. The results revealed a novel missense variant c.2530G > T (GCC → TCC), which was not observed in the 200 people controls, in WFS1 gene and cosegregated with hearing impairment in this family (Figure 3(a)). The variant c.2530G > T (p.Ala844Ser) was not listed in the public databases of HGMD, dbSNP, gnomAD, 1000 Genomes, and HapMap. Conservation analysis (Figure 3(b)) showed that the amino acid residue of this missense variant is conserved among 8 species (i.e., *Homo sapiens*, *Pan troglodytes*, *Bos taurus*, *Canis familiaris*, *Rattus norvegicus*, *Mus musculus*, *Gallus gallus*, and *Danio rerio*). Finally, the mutation p.Ala844Ser was predicted to be deleterious by MutationTaster, PolyPhen-2, LRT, and Fathmm software (Table 2).

**Figure 4:**
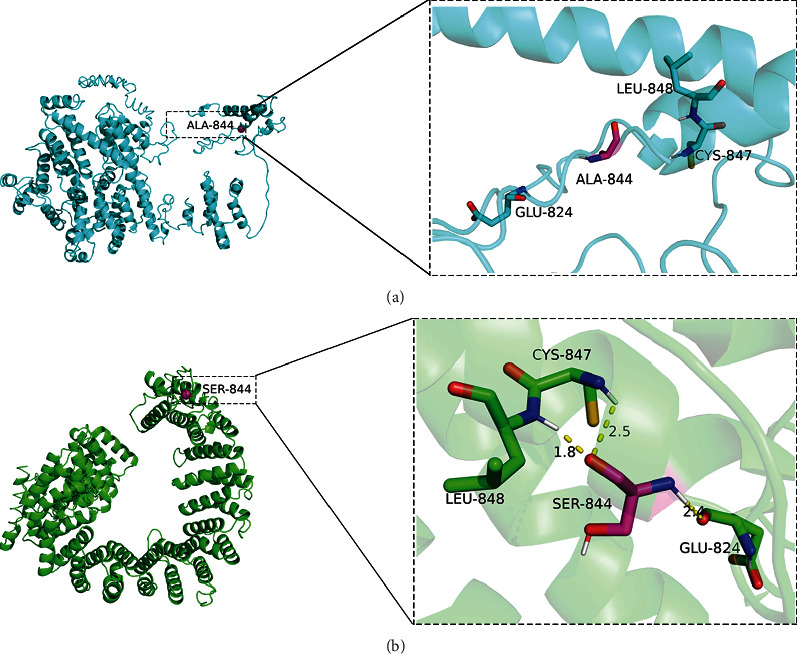
The 3D protein structural modeling comparison of wild-type (WT) and mutant WFS1. (a) Molecular modeling of WT WFS1. The black dotted box indicates the position of amino acid residue 844 of WT WFS1. (b) Molecular modeling of mutant WFS1. Hydrogen bonds linking Ser844 with Glu824, Ser844 with Cys847, and Ser844 with Leu848 (yellow dotted lines) are shown.

Based on the amino acid sequence of WFS1, the three-dimensional structures of WT and mutant WFS1 proteins were simulated. This missense variant caused a change from a nonpolar uncharged alanine (Ala) to a polar uncharged serine (Ser) at codon 844. The amino acid at position 844 in the WT protein structure showed no interaction with other amino acids analyzed by PyMOL software (Figure 4(a)). The substitution of WFS1 p.Ala844Ser could lead to the formation of two hydrogen bonds between the hydroxyl functional group of Ser844 and the N atoms of Cys847 and Leu848 at distances of 2.5 Å and 1.8 Å, respectively. Meanwhile, the N atom of Ser844 could interact with the Glu824 at distance of 2.4 Å (Figure 4(b)). These changes in amino acid interactions are predicted to prompt the formation of protein alpha helical structures.

### 3.3. Subcellular Localization of WFS1 Protein in HL Patients

**Figure 5:**
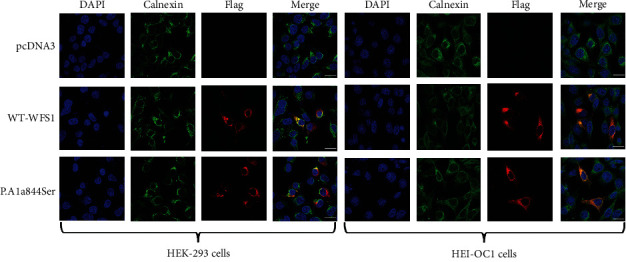
Subcellular localization of wild-type (WT) and mutated WFS1. Immunofluorescence staining was performed after transient transfection in HEK-293 cells and HEI-OC1 cells. Images display DAPI in blue, endoplasmic reticulum-specific marker Calnexin in green, Flag-tagged protein in red, and merged pictures. In both HEK-293 cells and HEI-OC1 cells, the variant protein was located in the cytoplasmic endoplasmic reticulum as in the WT. Scale bars =30 *μ*m.

In order to identify the intracellular location of the dominant variant, we constructed both the WT and mutant (p.Ala844Ser) WFS1 expression plasmids fused with Flag tags (Supplementary Figure 1). Then, we immunostained both HEK-293 cells and HEI-OC1 cells that transiently expressed WT and dominant variant p.Ala844Ser, respectively. The results showed that similar to WT, the wolframin protein bearing this variant was localized to the endoplasmic reticulum (ER) in both cell lines (Figure 5).

## 4. Discussion

*WFS1* is located in the 4p16.1, containing a total of eight exons, with exon 1 as a noncoding region and exon 8 encoding the large portion of the transmembrane regions and the carboxy terminals of the wolframin protein, which is a type of glycoprotein with nine transmembrane domains [12–14]. Pathogenic variants in the *WFS1* gene are generally associated with Wolfram syndrome (WS), Wolfram-like syndrome (WLS) and LFSNHL [15]. WS, also known as DIDMOAD, is an uncommon monogenetic spectrum disorder characterized by juvenile onset diabetes, diabetes insipidus, optic atrophy, and HL and often accompanied by other progressive neurological abnormalities [16]. The WS-induced deafness is recognized as HL at middle and high frequencies, and variants in *WFS1* are generally distributed throughout its coding region [17]. In contrast, the LFSNHL caused by *WFS1* variants at the DFNA6/14/38 locus is characterized by showing the bilateral symmetrical low frequency HL with the variants mainly located at the amino acids in the C-terminal domain of wolframin proteins [15].

In the present study, we identified the genetic variant responsible for the deafness phenotype in the pedigree of a five-generation Chinese family. The next-generation sequencing and sanger sequencing revealed a *WFS1* c.2530G > T missense variant that was cosegregating with HL in this family. Fukuoka et al.'s study showed that *WFS1*-associated LFSNHL was noticed mainly between 5 and 14 years old (average of 10 years old) and thereafter gradually progressed over a long period [8]. Thorpe et al.'s study further showed that *WFS1*-associated LFSNHL progressed to high-frequency HL, reaching at least a severe HL (>70 dB HL) at high frequencies by age 70 years [18]. Our findings in this study further supported these findings. The distribution plot of age at onset in this family (Figure 2) showed that the age of onset in WFS1 variant carriers was mainly concentrated between 6 and 15 years old. The PTA revealed a progressive HL in this Han family, in which only mild low-frequency HL was observed in 5-year-old patients, while the 70-year-old affected person developed with severe full-frequency HL. Notably, the result of Sanger sequencing showed that there was a heterozygous mutation in the 10-year-old child (V-8) of this family, but the PTA detection was normal (Supplementary Figure 2). We speculated that the child did not have a deafness phenotype because he had not reached the age of onset. In addition, none of the family members showed symptoms of tinnitus and vertigo, which was consistent with the results previously reported [15].

**Figure 6:**
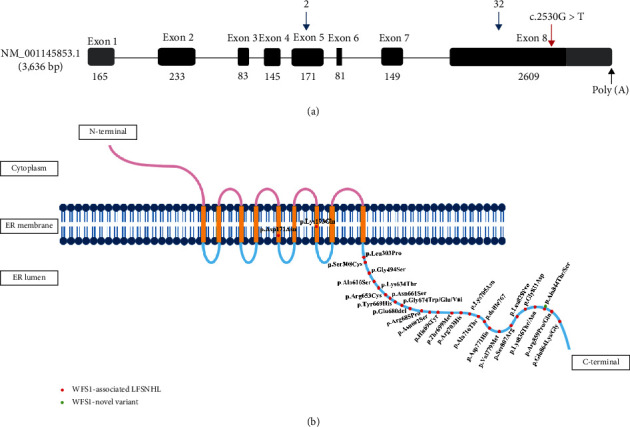
Variants of WFS1. (a) The position of *WFS1* c.2530G > T (p.Ala844Ser) highlighted with a red arrow. The gray region indicates the noncoding region; the blue arrows indicate the total number of variants reported in exons for *WFS1*-associated LFSNHL. (b) Overview of the reported WFS1-associated LFSNHL variants and their locations in the protein structure. The red dots indicate reported variants; the green dots indicate novel variant found in this study.

The results of spatial structure model analysis showed that the variant of p.Ala844Ser promoted the formation of hydrogen bonds between serine residue and multiple amino acid residues, resulting in the misformation of the *α*-helix structure at the C-terminal of wolframin protein. Hydrogen bond, as an intermolecular force, plays an important role in maintaining the spatial conformation and stability of proteins. Therefore, the formation of local abnormal conformation may interfere with the normal function of wolframin protein, e.g., disrupting the interaction between wolframin protein and other molecules or proteins. Alterations in the spatial structure and function of wolframin protein may be responsible for HL in this Chinese family. To date, there have been seven reported cases of LFSNHL in Chinese pedigrees caused by missense variants in *WFS1* (Table 3). Notably, a total of 34 known variants associated with LFSNHL are predominantly located in exon 8, except for two missense variants in exon 5 (Figure 6(a)), and most of the known variants in LFSNHL are concentrated in the C-terminal domain of wolframin protein (Figure 6(b)), indicating that the C-terminal domain of wolframin protein plays an essential role for the normal function of wolframin protein [35]. The wolframin protein mainly located in the ER widely exists in brain tissue, pancreatic *β*-cell, heart, lung, and placenta [36, 37]. Regarding the protein expression in the inner ear, studies reported that the expression of wolframin protein was observed in different cell types of mice and adult marmoset, including the organ of Corti, spiral ganglion neurons, and supporting cells of the cochlea [38, 39]. Morikawa et al. showed that both wild-type and mutant WFS1 (i.e., p.N325_I328del, p.Q194X, and p.L543R) were expressed in the ER of HEK-293 cells, which was consistent with our fluorescence results [40]. To observe the localization of variant wolframin protein in mouse cochlear hair cells, we performed immunofluorescence staining on HEI-OC1 cells and found that the results of HEI-OC1 cells were consistent with those of the HEK-293 T cells. Notably, the variants described by Morikawa et al. caused WS and resulted in significantly lower levels of variant protein expression in HEK-293 T cells than in WT [40], whereas the variant found in this study did not induce significant changes in wolframin protein levels in cells (Supplementary Figure 1). In addition, the WFS1 variants (i.e., p.N325_I328del, p.Q194X, and p.L543R) induced constitutive ER stress and cell apoptosis in HEK-293 T cells [40]. However, the mechanism of LFSNHL caused by WFS1 variants is still unclear. Because the HEI-OC1 cells themselves could highly express wolframin protein, considering that if the mutant plasmid is directly transfected into HEI-OC1 cells, the endogenous wolframin protein will compensate the molecular changes caused by the mutant plasmid. Therefore, to further explore the effect of wolframin protein bearing this variant on ER function in inner ear hair cells, the constructions of cell and transgenic mouse models carrying this variant are necessary.

The inheritance pattern of *WFS1* variants as well as the type of variants inherited could affect both the onset and the severity of the main clinical features of WS [41]. The *WFS1* variants are either homozygous or compound heterozygous in autosomal recessive hereditary WS. Studies have reported that the earlier-onset diabetes and optic atrophy occur in patients who are homozygous or compound heterozygous for two inactivating variants [42, 43]. Furthermore, compound heterozygosity for missense variants may lead to a mild phenotype [43]. However, patients who are compound heterozygous are at a higher risk of psychiatric disorders, DM, and HL [44]. The *WFS1* variants are heterozygous in autosomal dominant LFSNHL, and younger patients are often asymptomatic or exhibiting mild HL only at low frequencies. The novel missense variant c.2530G > T (p.Ala844Ser) identified in this study caused only nonsyndromic HL in this family. Interestingly, the variant with the substitution of A described in another independent family of nonsyndromic LFSNHL by Noguchi et al. [31] is not only in the same codon but also in the same genomic position as the variant with the substitution of T detected in our study, evidently suggesting that the Ala844 residue of WFS1 may play an important role in NSHL. To date, neither the type nor location of the pathogenic variant could predict the phenotype [45]. Therefore, the establishment of the genotype-phenotype correlations is extremely difficult due to the molecular complexity of WFS1, its diverse clinical features, and the small size of the patient cohorts.

In summary, we identify a novel heterozygous c.2530G > T *WFS1* variant cosegregating with HL in a five-generation Chinese family by targeted capture sequencing, which expands the mutational spectrum of *WFS1*. Our data provide additional molecular and clinical information for establishing a strong genotype-phenotype correlation for LFSNHL.

## Figures and Tables

**Table 1:** Summary of clinical data for members in the Chinese pedigree examined for genetic variants.

Subject	Gender	Age at test (years)	Age at onset (years)	Use of aminoglycoside	PTA (dB) right ear	PTA (dB) left ear	Tinnitus	Vertigo	Level of hearing impairment
II-7	F	70	30	No	68	66	No	No	Severe
III-6	F	55	21	No	60	60	No	No	Moderate
III-8	M	58	13	No	59	68	No	No	Severe
III-10	M	60	17	No	64	68	No	No	Severe
III-14	F	55	16	No	74	75	No	No	Severe
III-16	F	53	9	No	74	75	No	No	Severe
III-20	F	42	14	No	44	41	No	No	Moderate
III-22	M	44	14	No	46	53	No	No	Moderate
IV-2	F	30	11	No	36	41	No	No	Moderate
IV-8	M	30	—	No	13	13	No	No	Normal
IV-10	M	35	12	No	60	58	No	No	Moderate
IV-12	M	30	7	No	74	71	No	No	Severe
IV-14	F	26	—	No	11	14	No	No	Normal
IV-22	M	30	12	No	71	66	No	No	Severe
IV-29	M	16	8	No	38	38	No	No	Moderate
V-1	F	6	3	No	40	38	No	No	Moderate
V-3	M	13	1	No	85	85	No	No	Profound
V-8	M	10	—	No	20	20	No	No	Normal
V-9	M	4	—	No	14	14	No	No	Normal
V-15	M	5	5	No	29	29	No	No	Mild

**Table 2:** Characteristics of WFS1 variant and disease-causing effects based on various medical databases.

Gene symbol	Nucleotide change	Type of variation	Gene subregion	Amino acid change	MutationTaster	PolyPhen-2	LRT	Fathmm	Pathogenicity
WFS1	c.2530G > T	Missense	Exon 8	p.Ala844Ser	Disease causing	Possibly damaging	Deleterious	Deleterious	VUS

*Abbreviations*. c: variation at cDNA level; p: variation at protein level; VUS: variant of uncertain significance.

**Table 3:** WFS1 pathogenic variants found in patients with nonsyndromic low-frequency sensorineural hearing loss.

Origin	Exon	Nucleotide change	Protein change	Type of variant	Inheritance pattern	Reference
Portuguese	E-5	c.511G > A	p.Asp171Asn	Missense	AD	[19]
German	E-5	c.577A > C	p.Lys193Gln	Missense	Sporadic	[20]
Japanese	E-8	c.908 T > C	p.Leu303Pro	Missense	AD	[15]
Japanese	E-8	c.923C > G	p.Ser308Cys	Missense	AD	[15]
Japanese	E-8	c.1480G > A	p.Gly494Ser	Missense	AD	[7]
Chinese	E-8	c.1846G > T	p.Ala616Ser	Missense	AD	[21]
Japanese	E-8	c.1901A > C	p.Lys634Thr	Missense	AD	[22]
Chinese	E-8	c.1957C > T	p.Arg653Cys	Missense	AD	[23]
Japanese	E-8	c.1982A > G	p.Asn661Ser	Missense	AD	[15]
Taiwanese	E-8	c.2005 T > C	p.Tyr669His	Missense	AD	[24]
Chinese	E-8	c.2020G > T	p.Gly674Trp	Missense	AD	[4]
Dutch	E-8	c.2021G > A	p.Gly674Glu	Missense	AD	[20]
Dutch	E-8	c.2021G > T	p.Gly674Val	Missense	AD	[20]
Chinese	E-8	c.2036_2038delAGG	p.Glu680del	In-frame indel	AD	[23]
Japanese	E-8	c.2045A > G	p.Asn682Ser	Missense	Sporadic	[15]
American	E-8	c.2054G > C	p.Arg685Pro	Missense	AD	[25]
Chinese	E-8	c.2086C > T	p.His696Tyr	Missense	AD	[26]
Dutch	E-8	c.2096C > T	p.Thr699Met	Missense	AD	[9]
Chinese	E-8	c.2108G > A	p.Arg703His	Missense	AD	[26]
German	E-8	c.2115G > C	p.Lys705Asn	Missense	AD	[27]
Canadian	E-8	c.2146G > A	p.Ala716Thr	Missense	AD	[9]
Dutch	E-8	c.2300_2302delTCA	p.delIle767	Deletion	AD	[20]
Swiss	E-8	c.2311G > C	p.Asp771His	Missense	AD	[28]
American	E-8	c.2335G > A	p.Val779Met	Missense	AD	[9]
UK	E-8	c.2419A > C	p.Ser807Arg	Missense	AD	[20]
American	E-8	c.2486 T > C	p.Leu829Pro	Missense	AD	[9]
US	E-8	c.2492G > A	p.Gly831Asp	Missense	AD	[20]
Japanese	E-8	c.2507A > C	p.Lys836Thr	Missense	AD	[29]
Dutch	E-8	c.2508G > C	p.Lys836Asn	Missense	AD	[30]
Japanese	E-8	c.2530G > A	p.Ala844Thr	Missense	AD	[31]
American	E-8	c.2576G > C	p.Arg859Pro	Missense	AD	[28]
American	E-8	c.2576G > A	p.Arg859Gln	Missense	AD	[32]
Dane	E-8	c.2590G > A	p.Glu864Lys	Missense	AD	[33]
Chinese	E-8	c.2591A > G	p.Glu864Gly	Missense	AD	[34]

## Data Availability

The data used to support the findings of this study are available from the corresponding author upon reasonable request.

## Abbreviations

AD: autosomal dominant.

## Supplementary Materials

Supplementary Materials Figure S1: western blotting analysis based on the proteins extracted from HEK-293 cells and HEI OC1 cells. Figure S2: sequence chromatograms and the pure-tone audiograms for V-8 patient. Table S1: summary of the 127 targeted deafness genes.
