# Supplementary figures and images for: A Novel Missense WFS1 Variant: Expanding the Mutational Spectrum Associated with Nonsyndromic Low-Frequency Sensorineural Hearing Loss
Source: Biomed Res Int. 2022 Oct 3;2022:5068869. doi: 10.1155/2022/5068869 (PMC9550458; doi:10.1155/2022/5068869)

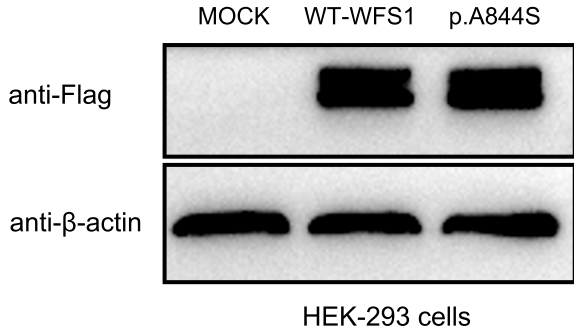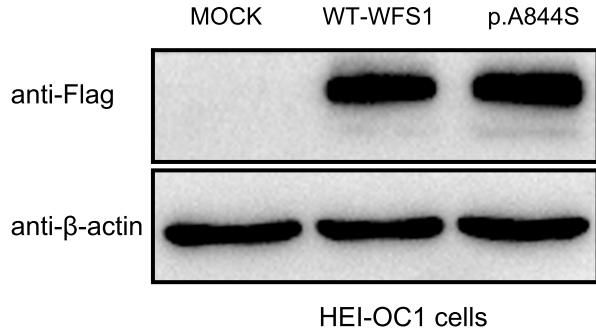

Supplement: Supplementary Materials — Figure S1: western blotting analysis based on the proteins extracted from HEK-293 cells and HEI OC1 cells. Figure S2: sequence chromatograms and the pure-tone audiograms for V-8 patient. Table S1: summary of the 127 targeted deafness genes. [file 5068869.f1.zip › Figure S1.pdf]

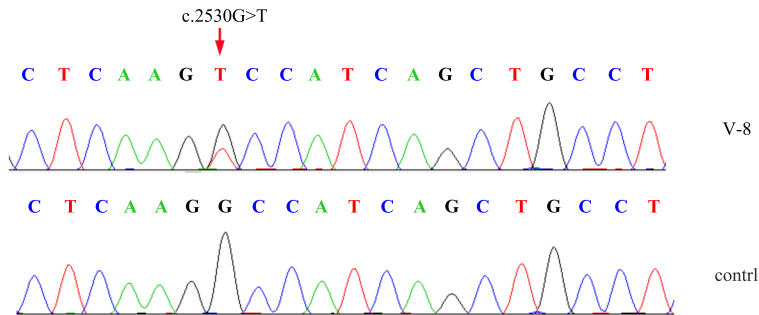

(a)

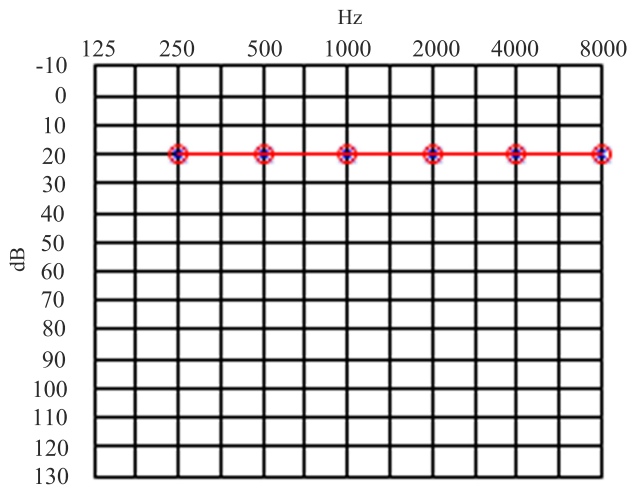

**V-8, male, 10y**

(b)

Supplement: Supplementary Materials — Figure S1: western blotting analysis based on the proteins extracted from HEK-293 cells and HEI OC1 cells. Figure S2: sequence chromatograms and the pure-tone audiograms for V-8 patient. Table S1: summary of the 127 targeted deafness genes. [file 5068869.f1.zip › Figure S2.pdf]
